# Localized Amyloidosis of the Nasal Mucosa: A Case Report and Review of the Literature

**DOI:** 10.3389/fsurg.2021.774469

**Published:** 2021-11-05

**Authors:** Hiromasa Takakura, Hirohiko Tachino, Kouji Takii, Johji Imura, Hideo Shojaku

**Affiliations:** ^1^Department of Otorhinolaryngology, Head and Neck Surgery, Faculty of Medicine, Academic Assembly, University of Toyama, Toyama, Japan; ^2^Department of Diagnostic Pathology, Faculty of Medicine, Academic Assembly, University of Toyama, Toyama, Japan

**Keywords:** localized sinonasal amyloidosis, calcification, ultrasonic bone curette, clinical features, debulking surgery

## Abstract

Amyloidosis is a disorder of protein folding in which various proteins automatically aggregate into a highly abnormal fibrillar conformation. Amyloidosis is classified into systemic and localized forms depending on whether the abnormal proteins deposited in several different organs or only a single organ. In localized amyloidosis of the head and neck regions, laryngeal amyloidosis is common; however, localized amyloidosis of the nose is extremely rare. We herein report a case of localized amyloidosis of the nose and review the relevant literature on localized sinonasal amyloidosis. A 41-year-old man presented with a history of severe nasal obstruction, which had persisted for two decades. Nasal endoscopy and imaging studies showed extensive thickening of the bilateral nasal mucosa and diffuse submucosal deposition of calcification. After histopathological and systemic examinations, he was diagnosed with localized amyloidosis of the nasal mucosa. Septoplasty and bilateral inferior turbinoplasty, which consisted of mucosal resection using an ultrasonic bone curette, was performed and his symptoms markedly improved. Localized sinonasal amyloidosis has a good prognosis and surgical resection should be selected as a first-line treatment; however, clinicians should recognize the high probability of recurrence.

## Introduction

Amyloidosis is a disorder of protein folding in which various proteins automatically aggregate into a highly abnormal fibrillar conformation (1). Amyloidosis is classified according to the type of protein deposited and can be either systemic (i.e., fibrils are deposited in various organs and tissues throughout the body) or localized (i.e., fibrils are produced only in one organ or site in the body) (2, 3). Presently, 18 proteins appearing as systemic amyloidosis and 22 as localized forms have been identified, and some proteins can appear as both systemic and localized amyloid deposits (4). The most common types are: AL (amyloid derived from the immunoglobulin light-chain) amyloidosis; AA (amyloid derived from serum amyloid A protein) amyloidosis, which is reactive amyloidosis due to chronic inflammatory diseases such as chronic infections and rheumatoid arthritis; ATTR (amyloid derived from the transport protein transthyretin) amyloidosis; and dialysis-associated amyloidosis (β2M type) (5, 6). Amyloidosis is a rare disease, with ~1,275–3,200 new cases per year occurring annually in the United States (1, 6). The most common subtype, AL amyloidosis has an incidence of 1 case per 100,000 person-years in Western countries (1, 6, 7).

Amyloid proteins can be deposited anywhere in the body (8). The deposition is extremely heterogeneous and the clinical presentation varies widely depending on which organs are involved (1). Amyloid can accumulate in the liver, spleen, kidney, heart, nerves, and blood vessels, causing different clinical syndromes, including cardiomyopathy, hepatomegaly, proteinuria, macroglossia, autonomic dysfunction, ecchymoses, neuropathy, renal failure, hypertension, and corneal and vitreous abnormalities (6). Systemic amyloidosis leads to serious signs and symptoms caused by progressive disease in organs and tissues (3). Localized amyloidosis is much rarer than systemic types, and as a result remains very poorly studied (9). Organ-specific localized amyloidosis can be found in Alzheimer's disease (β-protein in the plaques) and diabetes mellitus type 2 (amylin in the islands of Langerhans) (3). Nodular localized amyloid is an incidental finding and can be present in the skin, eyelid, conjunctiva, breast, larynx, bronchial tree, lungs, and genitourinary tract (3). Surgery is usually the treatment of choice in localized amyloidosis (3).

Localized amyloidosis in the head and neck region is a rare entity (10), and localized amyloidosis of the nose is extremely rare. Among localized amyloidosis foci in the head and neck region, the larynx is the most frequently involved site (61%), followed by the oropharynx (23%), trachea (9%), orbit (4%), and only 3% of cases are reported to involve the nasopharynx and sinonasal tract (11, 12). In 2019, only 19 cases of truly idiopathic primary localized sinonasal amyloidosis were documented (7).

We herein report a case of isolated localized AL amyloidosis of the bilateral nasal mucosa that was treated with debulking surgery using an ultrasound bone curette (UBC) and review the relevant literature on localized sinonasal amyloidosis.

## Case Report

**Figure 1:**
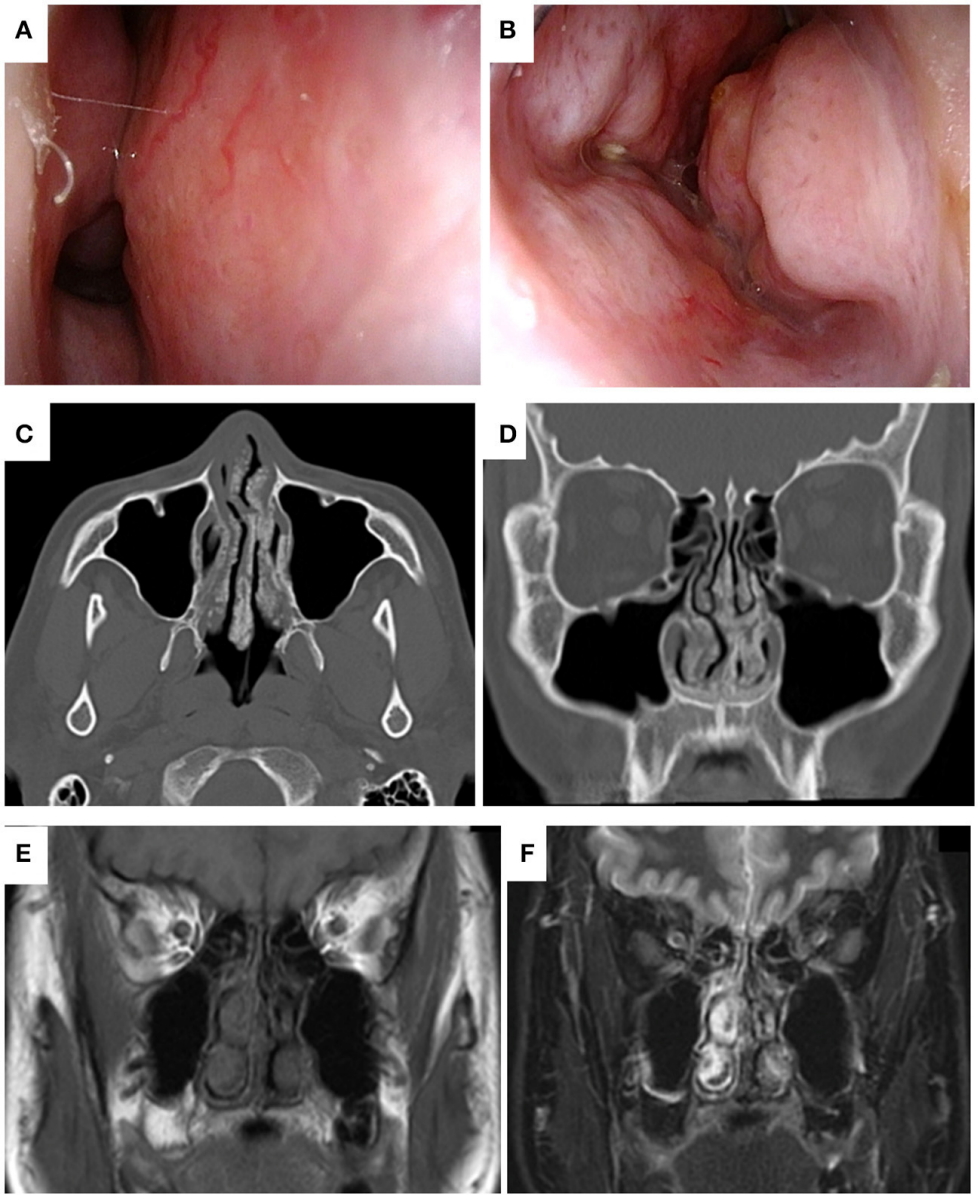
Endoscopic findings of localized sinonasal amyloidosis viewed from the right **(A)** and left **(B)** nasal cavities at the first visit to our hospital. The deviation of nasal septum to the right. The mucosa was smooth and pink, with nodular thickening, and bilateral narrowing of the common nasal meatus was observed. Plain computed tomography (CT) of the present case obtained at the previous hospital. Axial **(C)** and coronal **(D)** views of bone window CT of the head showed the deviation of the nasal septum to the right, thickening of the bilateral nasal mucosa, and deposition of microcalcified material in the submucosa of the nasal cavity, including the bilateral nasal septum, and middle and inferior turbinates. Magnetic resonance images of the present case obtained at the first visit to our hospital. Coronal views of plain T1-weighted **(E)** and T2-weighted imaging **(F)** of the head showed bilateral thickening of the nasal mucosa and numerous hypointense punctate signal areas in the submucosa of the entire nasal cavity.

**Figure 2:**
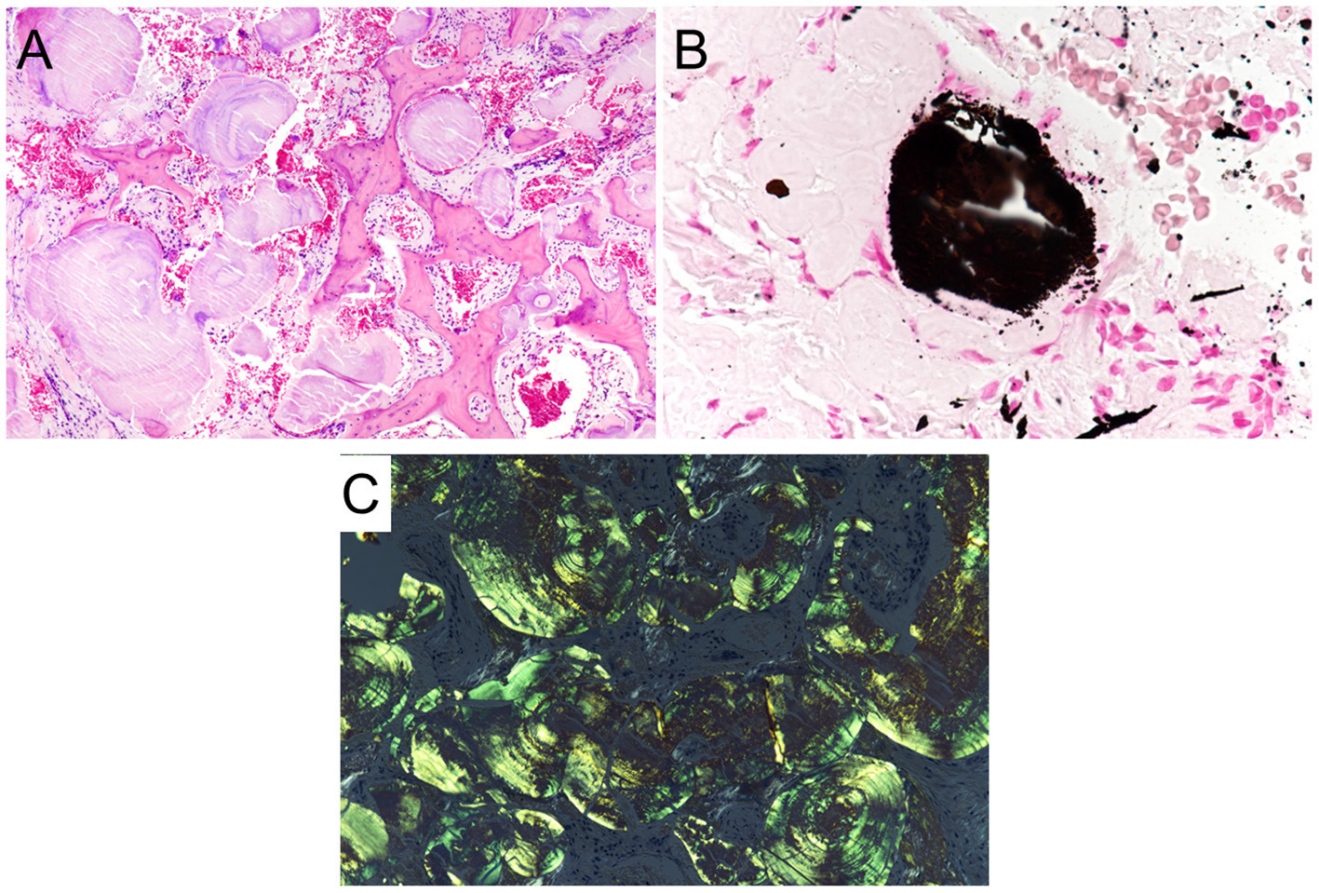
Histopathological features of localized amyloidosis of the nasal mucosa. **(A)** A histological examination revealed large deposits of amorphous eosinophilic with calcification (H&E staining, ×20). **(B)** The calcification was stained black (von Kossa staining, ×40). **(C)** Amyloid depositions with apple-green birefringence were found in the stroma when viewed with polarized light (Congo red staining, ×20).

A 41-year- old man with a history of atopic dermatitis and allergic rhinitis visited our hospital with a complaint of severe nasal obstruction that had gradually worsened since childhood. He also suffered from hyposmia and watery nasal discharge. On nasal endoscopy, the nasal septum was deviated to the right, the nasal mucosa was smooth and pink with nodular thickening, and the common nasal meatus was so narrow that the bilateral middle turbinates or posterior part of the nasal cavity were not visible (Figures 1A,B). Plain computed tomography (CT) revealed the deviation of the nasal septum to the right, thickening of the bilateral nasal mucosa, and deposition of microcalcified material in the submucosa of the nasal cavity, including the bilateral nasal septum, middle, and inferior turbinates (Figures 1C,D). Non-enhanced MRI showed bilateral thickening of the nasal mucosa and numerous hypointense punctate signal areas in the submucosa of the entire nasal cavity on both T1- and T2-weighted images (Figures 1E,F). An excision biopsy of the left inferior turbinate was undertaken on the day of the first visit to our hospital. A histological examination revealed large deposits of amorphous eosinophilic substance with calcification (Figure 2A), which was stained black by von Kossa staining (Figure 2B), and the presence of amyloid on Congo-red staining with apple-green birefringence when viewed with polarized light (Figure 2C). Immunohistochemistry was positive for serum amyloid L protein and kappa immunoglobin light chain. The patient was referred to the department of internal medicine in our hospital, where he was assessed for systemic disease. A complete blood count (CBC), and liver and renal function tests revealed normal results, and serum and urine electrophoresis was negative for Bence Jones protein and M protein. Duodenal and rectal biopsy using digestive tract fiberscopes did not show the deposition of amyloid in the tissue, and a bone marrow biopsy from the pelvis was negative for multiple myeloma. Echocardiography and electrocardiography revealed a normal heart function. Based on these findings, the final diagnosis was localized amyloidosis (AL kappa type) of the nasal mucosa.

**Figure 3:**
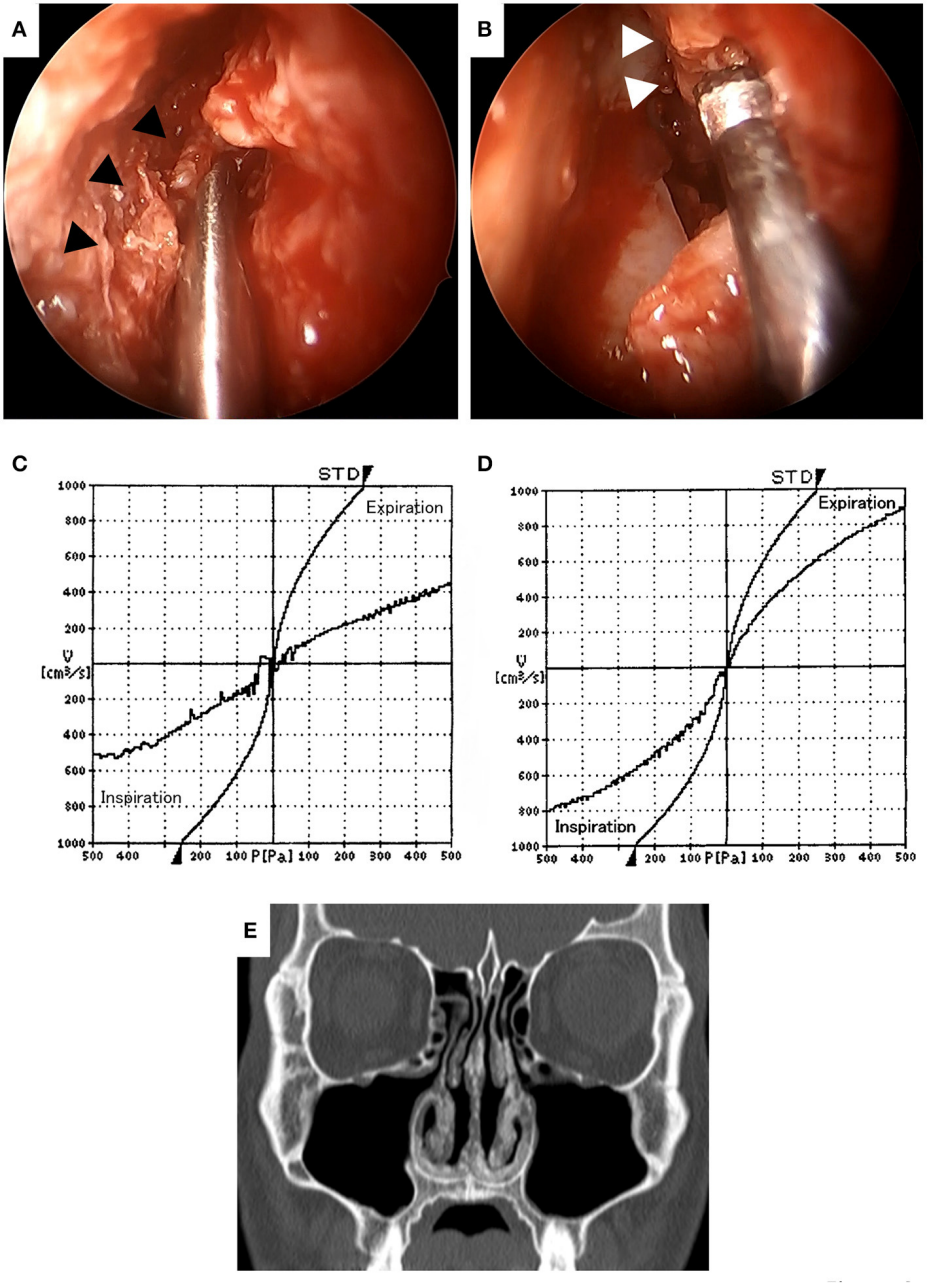
Intraoperative findings **(A,B)**. The submucosal tissue with severe calcification (▲) was very hard and difficult to resect with the turbinate blade of a microdebrider **(A)**. Hard submucosal tissue with calcification (Δ) was resected with an ultrasonic bone curette to preserve as much of the mucosal surface as possible **(B)**. Comparison of preoperative **(C)** and postoperative **(D)** rhinomanometry results at 6 months. The nasal resistance in both nostrils was improved after surgery. Computed tomography at 6 months after surgery **(E)**. Deviation of the nasal septum, mucosal thickening of the septum and bilateral inferior turbinates was improved and the bilateral common nasal meatuses were enlarged.

In order to improve his main symptom of nasal obstruction, septoplasty and bilateral inferior turbinectomy were performed at 4 months after the patient's first visit to our department. In the inferior turbinectomy, we applied the submucosal resection with powered instrumentation to preserve the ciliary function of the mucosa and to prevent postoperative crusting and bleeding. A mucosal incision was made in the anterior head of the inferior turbinate, the mucosa on the sides of the common and middle nasal meatus was detached from the bone, and submucosal resection was performed to remove as much thickened submucosal tissue as possible using a microdebrider. The bone of the inferior turbinate was not removed in consideration of the possibility of reoperation for recurrence of the lesion in the future. In septoplasty, the Killian approach with cartilage preservation was used and the deviated perpendicular plate of ethmoid bone and vomer were resected. The thickened submucosal tissues of the bilateral septal mucosa were reduced in the same manner as in the inferior turbinectomy. Since the submucosal tissue with severe calcification was very hard and difficult to resect with the turbinate blade of a microdebrider (Figure 3A), an ultrasonic bone curette (UBC) (Sonopet®) was also used to partially resect the submucosal tissue by shaving it off (Figure 3B). The use of these two devices allowed us to enlarge the bilateral nasal meatus, albeit partially, while preserving some of the mucosal surface. No surgical manipulation of the external nose or nasal septum cartilage was performed based on the patient's wishes. There were no complications during surgery. To prevent postoperative mucosal adhesion and dryness, one 0.5-mm thick silicone plate was inserted into each nasal cavity, sutured to the mucosa of the nasal septum, and removed after 1 month. Subjective nasal obstruction was assessed using the Nasal Obstruction Symptom Evaluation (NOSE) scale (13) and a visual analog scale (VAS) pre and postoperatively. A VAS was a horizontal line, 100 mm in length, anchored by the word “No nasal obstruction at all” at the left end and by the word “Complete nasal obstruction” at the right end, and we asked the patient to mark the point that represented his perception of his current state on the line. The VAS score was determined by measuring in millimeters (mm) from the left end of the line to the point that the patient marks. At 6 months postoperatively, the symptoms of nasal obstruction had markedly improved and there were no significant post-operative symptoms. The NOSE scale improved from 8 points preoperatively to 3 points postoperatively on a 20-point scale and the VAS score improved from 72 mm preoperatively to 25 mm after surgery. The objective evaluation of nasal obstruction using rhinomanometry also revealed the improvement of nasal resistance in both nostrils (Figures 3C,D). CT revealed the enlargement of the bilateral common nasal meatus (Figure 3E). At 8 months after surgery, there was no recurrence or progression of symptoms. The patient is currently being followed up and is satisfied with the improvement of nasal obstruction and the absence of symptoms after surgery.

## Review of the Relevant Literature on Localized Sinonasal Amyloidosis

We only identified 21 case reports of localized amyloidosis originating from the nasal cavity and paranasal sinuses that included detailed patient information in 20 English-language articles published from 1990 to date (7, 14–32). The clinical characteristics of the reported cases localized sinonasal amyloidosis, including our own, are shown in Table 1. Cases of localized amyloidosis originating from the nasopharynx were excluded from this review.

**Table 1:** Summary of clinical characteristics of previously reported localized sinonasal amyloidosis.

	**Age**	**Sex**	**Symptoms**	**Duration of symptoms**	**Location**	**Side**	**Past history**	**Family history**	**Local examination findings**	**CT**	**MRI**	**Treatment**	**Recurrence or progression of the disease**	**Type of amyloid protein**	**Follow-up**
Mufarrij et al. (14)	8	M	Nasal obstruction. Mouth breathing. Recurrent epistaxis. Frontal headache.	1 y	Bil nasal cavity and ESs.	B	Recurrent ear discharge	N.D.	Marked Bil IT hypertrophy completely obstructing the nasal airways. Irregular mass arising from the roof of the nasopharynx.	A lobulated enhancing mass lesion centered in the nasal cavities and ESs with complete opacification of the MS and SSs.	N.D.	Ethmoidectomy and biopsy	+	AL λ	15 mo.
Birchall et al. (15)	38	F	Painless facial swelling.,	2 y	R MS and antrum	R	No	N.D.	A swelling overlying the R maxilla with protrusion through the roof of the mouth.	A heterogeneously enhancing soft tissue mass arising from R maxillary antrum with erosion and expansion of the bony walls of the sinus. Extension through the orbital floor and into the nasal-cavity, nasopharynx, FSs and ESs, and soft tissues of the cheek.	T1-weighted images: a large mass of mixed signal intensity containing areas of both intermediate and low signal intensity. T2-weighted images: high signal intensity of the periphery of the mass right low signal intensity of the central part of the lesion.	Hemi-maxillectomy and reconstruction	N.D.	Negative for κ and λ light chains	N.D.
Pang et al. (16)	10	F	Nasal obstruction. Nnasal discharge. Mouth breathing. Epiphora. Occasional epistaxis.	1 y	R IT	R	N.D.	N.D.	An erythematous polypoidal mass arising from the R IT. The mass appeared friable, with irregular waxy gray or yellow mucosa filling the entire R nasal cavity.	Bil IT masses with osteotic changes.	An enhancing mass arising from the R IT and Bil retention polyps in the MSs	Diagnostic surgery	+	N.D.	3 y
Tsikoudas et al. (17)	53	F	Discomfort over the maxillary sinuses. Nasal blockage. Nasal discharge	2 y	Nasal septum. R lateral wall, R antrum, R ES	R	N.D.	N.D.	A large lesion affecting the nasal septum and R lateral nasal wall that was friable and bled with mild trauma.	The lesion involved the R nasal cavity, with lateral erosion involving the anterior ethmoid cells	A large lesion affecting the nasal septum and R lateral nasal wall that was friable and bled with mild trauma	Debulking biopsy	–	AL	3 y
Chin et al. (19)	21	M	Nasal stuffiness, Dysphonia	1 y	Bil nasal conchae and the adjacent osteomeatal infundibulum Bil ESs and MSs.	B	N.D.	N.D.	Whitish, ulcerative, and swollen nasal conchae.	The affected bony conchae and sinus walls had a “fluffy” and somewhat hyperplastic reaction adjacent to the soft tissue mass that filled both nasal fossae and the ESs	The lesion had low to intermediate signal intensity on both T1- and T2-weighted images and demonstrated peripheral enhancement on the contrast-enhanced study. Typical of obstructive secretions within the paranasal sinuses	Surgical excision	N.D.	N.D.	N.D.
Ali et al. (20)	48	M	Cerebrospinal fluid leak from the L nostril	N.D.	The sphenoid body and SS extending posteriorly into the clivus	L	N.D.	N.D.	CSF leak and a soft mass in the L SS.	Soft tissue mass in the region of the sphenoid body and SS extending posteriorly into the clivus associated with destruction of bones in this region. Bony defect in the floor of the pituitary fossa, sphenoid sinus and roof of the nasopharynx.	N.D.	Surgical excision	-	N.D.	12 m
Prasad et al. (21)	42	F	Recurrent L nasal obstruction Recurrent epistaxis	4 y	L nasal cavity	L	Diabetics	N.D.	Significant reduction of the L nasal airway. A smooth, solitary, soft to firm, non-tender, pale pink mass between the septum and the lateral wall above the IT.	N.D.	A soft tissue mass in the L nasal cavity involving L IT and retained secretions in the L MS without bone erosion.	Endoscopic removal of the mass	-	N.D.	8 mo
Teo et al. (18)	18	F	R nasal blockage Epiphora	1 y	R IT extended from the nasopharynx to Bil posterior nasal cavities.	R	N.D.	N.D.	A large R IT mass.	Recurrence in the nasal cavity, osteitic changes, and fluid in the MSs and SSs.	Enhancement of the R IT mass.	Inferior turbinectomy	+	N.D.	4 y
Sass et al. (22)	46	M	Nasal obstruction Rhinorrhea, Maxillary and frontal sinusopathy L auricular plenitude. Recurrent epistaxis	3 y	Medial wall, nasal infundibula, nasal septum, and MS	L	N.D.	N.D.	Lesion in the L nasal meatus, soft palate convexity A light retraction of the L TM (tympanic membrane)	An obliteration by material with density of soft parts and an expansive effect of the MS and nasal meatus to L, Erosion of the maxillary sinus medial wall, nasal infundibula and nasal septum. The MS reactive thickening.	N.D.	Endoscopic resection	-	N.D.	3 y
Pearlman et al. (23)	55	F	Severe malaise Diffuse arthralgias Nasal congestion	N.D.	The nasal and paranasal mucosa R and L MSs. nasal septum, and Bil ITs	B	Chronic sinusitis Rheumatoid arthritis Monoclonal gammopathy	N.D.	A nasal polyp arising from the L middle meatus.	An opacification of L middle meatus and mucosal inflammation in all sinuses.	N.D.	N.D.	N.D.	N.D.	N.D.
Nakayama et al. (25)	14	F	Anosmia. Facial pain. Recurrent Bil epistaxis	2 y	The nasal mucosa	B	N.D.	N.D.	Erythematous mass completely obstructing the Bil middle meatuses and olfactory clefts	Complete opacification of all the sinuses	T2-weighted magnetic resonance (MR) imaging: hypointense lesion indicating amyloidosis and a hyperintense lesion indicating entrapped secretions in the sinuses around the hypointense lesion	Surgery of nose, ESS	+	AL	4 y
Nakayama et al. (25)	27	F	Bil impaired hearing Recurrent Bil epistaxis.	N.D.	The nasal mucosa of Bil ITs	B	N.D.	N.D.	Serous effusion in the ear An erythematous mass in the regions of the Bil ITs	Irregular hypertrophy of the Bil ITs.	N.D.	Observation	N.D.	AL	N.D.
Naidoo et al. (24)	50	F	Nasal obstruction, Hyposmia Post-nasal drip	Several years	The nasal cavity and ES	B	A septoplasty and bilateral inferior turbinoplasties	N.D.	Significant adhesions between the septum and the lateral nasal wall Thickened mucosa overlying the MT with total obliteration of the space medial to the MT.	Disease restricted to the nasal cavity and ESs. Mucosal thickening along the roof of the nose.	N.D.	Dissection of affected tissue	+	N.D.	6 w
Cunningham et al. (26)	67	F	Nasal congestion. Epistaxis.	1 y	Nasal mucosa	L	Nasal trauma	N.D.	L sided nasal obstruction caused by grade 3 fleshy polyps extending into the post-nasal space.	L sided polyposis containing areas of calcification.	N.D.	Debulking and ESS	+	non-AA	6 mo
Rauba et al. (27)	53	F	Nasal stuffiness, Rhinorrhea, Loss of smell, Bil epistaxis. Bil proptosis. Slight vision impairment. Severe facial pain.	2 y	Bil middle meatus. The olfactory clefts, The ES, L MSs,FSs,SSs. A marked deformation and erosions of L orbital wall with a dislocation of the intra-orbital structures	B	No allergies.	No	Nasal polyps arising from both the middle meatus. Some edematous masses on the posterior edge of the R IT. Erythematous masses completely obstructing the Bil middle meatus and the olfactory clefts Proptosis of both eyes, greater on the left side	A complete opacification of all the sinuses.	Multiple inhomogeneous masses with septations, involving the ethmoidal cells with a predominant expansion to the L MS Fs and SS. A marked deformation and erosions of the L orbital wall with a dislocation of the intraorbital structures Secretions and hypertrophic mucosa within the paranasal sinuses Only a partial septal and peripheral enhancement in the masses	Ethmoidectomy with the enlargement of the natural ostium for all the sinuses Total ethmoidectomy, Draf type II A and Bil maxillectomy (type II)	+	AL	18 mo
Doshi et al. (28)	70	F	Chronic R nasal obstruction R epiphora.	N.D.	R IT. R lateral nasal wall, corresponding to the orifice of the nasolacrimal duct.	R	Radiation therapy for a nasal cavity lymphoma. 2 surgical procedures for occluded nasolacrimal duct.	N.D.	A smooth, pink, friable mass abutting the posterior aspect of R IT and extending to fill most of the nasopharynx. An erythematous irregular area, smaller than 1 cm, on the R lateral nasal wall, corresponding to the orifice of the nasolacrimal duct.	A partially calcified mass in the R inferior meatus	The mass, seen prolapsing into the nasopharynx, to be slightly hypointense relative to skeletal muscle on T1-weighted image without contrast. The hypointense mass on coronal T2-weighted and contrast-enhanced, fat-saturated T1-weighted images.	N.D.	N.D.	N.D.	N.D.
Kumar et al. (29)	55	M	Bil nasal obstruction, Recurrent nasal bleeding. Bill hearing loss.	7 y	Nasal cavity. Nasal septum Sella and supra seller region Medial wall of MS., Hard palate. Pterygoid process on both side.	B	N.D.	N.D.	Bil pale pink, non-tender, and soft to firm masses between the septum and the lateral wall above the IT.	Enhancing mass extending from nasal cavity to sella and supra seller region with destruction of medial wall of MS, nasal septum, hard palate, pterygoid process on both sides	N.D.	Conservative surgery.	N.D.	AL (Both of theλandκ)	N.D.
Nishimura et al. (30)	60	F	Nasal blockage. Discomfort around the eyes. Swelling of the upper R eyelid and eye pain. Headache.	8 y	Bil nasal cavity Sinuses. L eye socket.	B	No	No	Masses accompanied by crusting. Partially yellow granular nodules under the mucosa. Intranasal adhesion.	A destructive and expansive tumor in the nasal cavity advancing across the sinuses with punctate calcification. The tumor extended into the L orbit,	N.D.	Chemotherapy (melphalan and dexamethasone). Extensive ESS.	–	AL-κ	18 mo
Wahid et al. (32)	61	M	L nasal obstruction Nasal discharge Recurrent epistaxis.	1 y	L IT	L	HT Obesity	N.D.	A friable exophytic mass originating from the posterior aspects of the Lt IT.	Thickening of the posterior aspect of the L IT. The opacification of the MS. Middle meatal occlusion by the mass. Subtle reactive bone changes within the L IT and lateral wall	Soft tissue density mass in the left maxillary sinus, which is hyperintense postcontrast enhancement and is seen closely abutting the L IT extending posteriorly into the nasopharynx on T2-weighted image. Soft tissue density mass in the L maxillary sinus, which is isointense to the adjacent skeletal muscle on T1-weighted image.	Eexcisional biopsy	–	negative for AA, ALκand Al λ	6 mo
Iliev et al. (31)	81	F	Difficulty of nasal breathing. Facial deformity.	Several months	Bil MTs and STs. Bil FSs. Bil nasal bones. Bil anterior ESs, R orbit.	B	No HT, DM	N.D.	A bulging mass engaging the nasal meatuses.	Heterogeneous mass, engaging the nasal meatuses at the level of the MT and STs, spreading along the FSs, with destruction of the base of the latest, An osteolysis of the nasal bones bilaterally and the anterior walls of the anterior ESs. A suspicion for the zone of destruction in the medial wall of R orbit.	N.D.	External beam radiotherapy (36 Gy)	–	N.D.	9 mo
Singh et al. (7)	14	M	Bil. nasal obstruction. Snoring, mouth breathing. Expansion of nasal dorsum region.	Since early childhood	Bil. nasal mucosa. Bil. FSs, ESs, MSs.	B	A nasal surgery for nasal obstruction, snoring and mouth breathing.	N.D.	Bulky septum. Non tender, firm, non-fluctuant with pale and edematous overlying mucosa. Hypertrophies of Bil ITs with polypoidal changes. Extensive synechiae between nasal septum and IT.	Severely restricted nasal passage with significantly thickened nasal septum and Bil ITs. Blockage of OMC and patchy mucosal thickening involving FSs, ESs, MSs.	N.D.	Excisional biopsy.	N.D.	N.D.	N.D.
Present case	41	M	Severe nasal obstruction Hyposmia Water nasal discharge	Since early childhood	Bil. nasal mucosa.	B	Atopic dermatitis Allergic rhinitis	No	Deviation of nasal septum to R. Bil nasal smooth, pink, nodular thickening of nasal mucosa. Bil narrowing of common nasal meatuses.	The deviation of the nasal septum to the R, The thickness of Bil nasal mucosa and deposition of microcalcified material in the submucosa of the nasal cavity	Numerous hypointense punctate signals in the submucosa of the entire nasal cavity on both T1- and T2-weighted images.	Septoplasty and bilateral inferior turbinectomy. Debulking surgery.	-	Alκ	8 mo

*Bil, bilateral; L, Left; R, Right; B, both; CSF, cerebrospinal fluid, OMC, osteomeatal complex; ST, superior turbinate; MT, medial turbinate; IT, inferior turbinate; N.D., not described; M, male; F, female; y, years; mo, months; w, weeks; HT, hypertension; DM, diabetics mellitus; λ, lambda; κ, kappa; MS, maxillary sinus; ES, ethmoid sinus; FS, frontal sinus; SS, sphenoid sinus*.

### Clinical Characteristics of Localized Sinonasal Amyloidosis

In the 22 case reports included in our literature review (Table 1), the mean age of patients with localized sinonasal amyloidosis was 42.4 years (range: 8–81 years). Eight of the patients were men and 14 were women; thus, there is a female predominance in the incidence of localized sinonasal amyloidosis.

Regarding the past medical history of the patients, 4 cases (18.2%) had no history and 3 cases (13.6%) had a history of nasal surgery. In addition, diabetes, hypertension, obesity, radiotherapy for a nasal cavity lymphoma, nasal trauma, rheumatoid arthritis, monoclonal gammopathy, and ear discharge were reported in one case each (4.5%). The past medical history was not described in 9 cases (40.9%) reported in 8 studies. Three cases (13.6%), including our case, had no relevant family history, and the remaining case reports did not describe the family history.

The clinical symptoms of the patients included nasal obstruction (including nasal difficulty, nasal blockage, nasal congestion or stuffiness) (*n* = 18; 81.8%), bleeding (including recurrent epistaxis, *n* = 9; 40.9%), rhinorrhea or nasal discharge (*n* = 7; 31.8%). Facial swelling and olfactory impairment (including anosmia or hyposmia) were found in 4 cases each (18.2%). Mouth breathing, epiphora and hearing impairment [including aural fullness, described as “auricular plenitude” in the case report by Saas et al. (22)], were found in 3 cases each (13.6%). Headache, facial pain, facial discomfort, or recurrent sinusitis (including maxillary and frontal sinusopathy) were found in 2 cases each (9.1%). Snoring, eye pain, proptosis with visual impairment, cerebral fluid leak from the nostril, severe malaise and diffuse arthralgia were found in one case each (4.5%).

The duration of symptoms was 1 year in 6 cases (27.2%), 2 years in 4 cases (18.2%), and since early childhood in 2 cases (9.1%). One patient each (4.5%) reported that the duration of symptoms was several months, 3 years, 4 years, 7 years, 8 years, and several years. The duration of symptoms was not described in 4 reports.

The locations of lesions included nasal mucosa (including septum, floor, middle or inferior turbinate) (*n* = 20; 90.9%), maxillary sinus or antrum (*n* = 8; 36.4%), ethmoid sinus (*n* = 6; 27.3%), frontal sinus (*n* = 3; 13.6%) and sphenoid sinus (*n* = 2; 9.1%). Extra-nasal or extra-sinus extension to the sella, parasellar region, clivus, hard palate, pterygoid process and nasal bone were found in one case each (4.5%) in 4 case reports.

The laterality of regions included bilateral lesions in 12 cases (54.5%), and right- and left-side regions in 5 cases each (22.7%).

Regarding the findings of local examinations, a mass in the nose was found in 14 cases (63.6%). The mass was characterized as erythematous (*n* = 4), pale pink or pale (*n* = 3), friable (*n* = 2), smooth (*n* = 2), soft to firm (*n* = 2), and non-tender (*n* = 2). Local examinations revealed adhesion (or synaechie) and nasal polyp in 3 cases each. Thickening of the mucosa of the nasal cavity, especially affecting the nasal septum and nasal conchae was reported in 5 cases (22.7%) and localized lesions in the nasal mucosa were reported in 2 cases, which were described as a partially yellow granular nodule (*n* = 1) and an erythematous irregular area (*n* = 1).

Regarding the diagnostic imaging studies, X-ray, CT, and MRI were used in 2 (9.1%), 21 (95.5%), and 10 (45.5%) cases, respectively. Of the 21 patients who underwent CT, all showed increased lesions of soft tissue density located in the nose or sinuses; findings included opacification, mass or mucosal thickening of the nasal meatus or sinus, and masses. Among them, calcification in the lesion was observed in 5 cases (23.8%), osteitic, osteotic, or reactive bone change were observed in 3 cases (14.3%), and destructive changes of the bone or osteolysis were observed in 4 cases (19.0%). MRI signal intensity of the amyloid legion was documented on T1-weighted images in five cases and T2-weighted images in six cases. For T1-weighted images, three of the five cases (60.0%) were hypointense and two were hypointense to isointense (40.0%); for T2-weighted images, three of the six cases were hypointense (50.0%), one was hypointense to isointense (16.7%), one was dominantly hyperintense (16.7%), and one was hyperintense in the periphery and hypointense in the center (16.7%).

Regarding the type of amyloid protein, 11 cases in 10 studies were subjected to an immunohistochemical examination. Among them, serum amyloid light protein (AL) was positive in 8 cases, including kappa immunoglobin light chain in 2 cases, lambda immunoglobin light chain in one case and both kappa and lambda in one case. The remaining 3 case reports described negative studies for AL, serum amyloid A protein (AA) and both (*n* = 1 each).

Seventeen cases (81.8%) were treated with surgery. Among them, 4 cases (18.2%) were treated bv diagnostic surgery or excisional biopsy alone. The remaining 13 patients underwent resection or debulking of the lesion, with sinus surgery performed as needed. In one case, chemotherapy (four 28-day cycles of melphalan [8 mg] and dexamethasone [40 mg/day] on days 1–4) was administered prior to surgery, but no reduction was seen (30). External radiotherapy was selected for a large destructive amyloid lesion in one case and the progression of the amyloid lesion was halted (31). Observation was selected in one case and the details of treatment were not described in 2 cases. Recurrence or the progression of the disease was confirmed in 7 of the 15 cases in which it was described (46.7%).

The mean follow-up period was 20.4 months (range, 1.5 months [6 weeks] to 48 months [4 years]).

## Discussion

In this latest review of the relevant literature, we aimed to clarify the clinical features of localized sinonasal amyloidosis. From an epidemiological point of view, our review of the literature revealed that localized sinonasal amyloidosis occurs in a wide age range (8–81 years), has a female predominance (8 males:14 females), and tends to not be associated with family history. Regarding past medical history, three of the cases had undergone nasal surgery prior to the diagnosis of amyloidosis, which suggests that patients with undiagnosed amyloidosis are mixed in among those undergoing nasal surgery to improve nasal symptoms. The most common clinical symptoms were nasal obstruction (81.8%), epistaxis (40.9%), and nasal discharge (31.8%), in that order, and there no symptoms were specific for localized sinonasal amyloidosis. However, there were some cases with symptoms suggestive of severe inflammation or malignancy, such as headache, facial pain, visual impairment or cerebral fluid leak from the nostril (15, 20, 29–31). The duration of symptoms up to the initial diagnosis was in the order of years for most patients, suggesting that the symptoms of localized sinonasal amyloidosis progress very slowly. There was no predominant laterality of the lesions.

On local examination, sinonasal amyloidosis tended to present as erythematous, pale pink or pink, friable, smooth-surfaced masses. Localized mucosal lesions showed a yellowish, granular, irregular mucosa. A certain number of non-operative or early postoperative cases showed mucosal adhesions, which were thought to be a feature of amyloidosis.

The imaging features of localized amyloidosis in the head and neck are not specific (33). On CT, our review indicated thickening of the sinonasal mucosa or formation of a soft-density mass, occasionally including calcification or osteotic change of the adjacent bone. Chin et al. reported a “fluffy” appearance around the sinonasal bones adjacent to the amyloid deposits as a possible representative finding suggestive of this disorder, because fluffy bone changes were not seen in other calcifying sinonasal diseases, including inspissated secretion, fungal mycetoma, cartilaginous tumor, and olfactory neuroblastoma (19). They suggested that the osteoblastic reaction to submucosal amyloid deposition in the submucosa may induce fluffy bone changes (19). In our case, there were few fluffy bony changes, and the image showed a lot of punctuate calcification deposited under the extensive nasal mucosa, which seemed to depict calcification of the amyloid material itself. The MRI signal intensity of amyloidosis can be widely variable, appearing similar to skeletal muscle on T1- and T2-weighted imaging (19). A previous study indicated that the MRI signal intensity of amyloidoma, which is a solitary mass of amyloid protein, is low-to-intermediate on T1-weighted imaging and intermediate-to-high on T2-weighted imaging (8). Our review of the relevant literature showed that the sinonasal amyloidosis had a low to intermediate signal intensity on T1-weighted MRI imaging, and varying signal intensity, mainly low but sometimes moderate to high intensity, on T2-weighted MRI imaging. Lee et al. hypothesized that varying MRI signal intensity could be caused by the density of the amyloid protein deposition (34).

In order to make a diagnosis of amyloidosis, the first step is to obtain histological evidence of amyloid, followed by looking for evidence of systemic amyloid deposition in the patient. The next steps are the detection and (in the case of AA and AL) quantification of the precursor proteins in the blood to confidently determine the type of amyloid (3). The presence of amyloid is proved by a tissue specimen that is positive for Congo Red staining, with characteristic apple-green birefringence under polarized light (3). Fat tissue stained with Congo red has high sensitivity (up to 90%) and high specificity (almost 100%) for AL, AA, and hereditary ATTR. The sensitivity for these types is ~80% in rectal tissue and ~60% for bone marrow in AL (3). If amyloid is detected in sites specific for localized amyloidosis (genitourinary tract, eyelid, conjunctiva, larynx, and so forth), checking for amyloid in other sites—such as fat tissue, rectum, bone marrow, or salivary glands—is recommended before diagnosing localized amyloidosis (3). In general, the prognosis for localized and systemic amyloidosis is quite different. Localized amyloidosis has a high survival rate (about the same as the general population), while systemic dissemination has a dismal average survival time of 5–15 months (10). It is very rare for localized amyloidosis to become systemic in the natural course of the disease, with only 7 of 606 cases (1%) showing this change in a previous study (9). Thus, differentiating between localized and systemic amyloidosis is critical to its management. Systemic amyloidosis is diagnosed when amyloid is present in two different body parts (3). In our case, histological evidence of amyloid deposits was only observed in the nasal mucosa, no amyloid deposit was observed in the duodenum, rectum, or bone marrow, and no abnormalities were found in other vital tissues (heart, liver, kidney, and blood), which finally led to the diagnosis of localized amyloidosis of the nasal mucosa.

After the detection of amyloid, the type of amyloid should be identified by the immunohistochemical analysis of biopsy samples using specific antibodies (3). AL is the most common subtype of amyloid precursor proteins in most organs (35), and AL is associated with the clonal proliferation of plasma cells, producing a monoclonal protein that circulates in the blood and amyloid fibrils that are derived from the immunoglobulin light chain or its fragments (5). AL lambda chains represent the majority of cases in systemic amyloidosis (2–4 times more frequent than AL kappa type) (36), whereas kappa type AL amyloidosis is as common as that of lambda type in localized amyloidosis, suggesting that the pathogenesis of the localized form is different from that of the systemic form (2). Our review of the literature indicated that AL was the most common type of amyloid protein (8/11 cases, 72.7%) in localized sinonasal amyloidosis. Of these, the ratio of AL kappa to AL lambda was 3:2, suggesting that the frequency of AL kappa type is approximately equal to that of AL lambda type in localized sinonasal amyloidosis. Previous studies support our results.

For localized amyloidosis of the head and neck region, symptom-based management is recommended. If systemic amyloidosis is ruled out by appropriate testing, treatment may include observation in the absence of symptoms or conservative resection for symptom relief followed by annual follow-up for up to 10 years (10). However, the risk of recurrence after conservative resection for localized amyloidosis is high. In a previous study that examined the natural history and outcome of localized amyloidosis in 606 patients, approximately half of all patients required only a single surgical intervention and a fifth of all patients required repeated interventions to improve their symptoms (9). In our review of the literature on localized sinonasal amyloidosis, surgical intervention for symptom relief was performed in ~60% of all cases, and the recurrence or the progression of symptoms was reported in approximately half of the cases for which the clinical course was described, which was similar to the results of previous studies.

Inferior turbinoplasty is an effective adjunctive procedure to septoplasty for patients with inferior turbinate hypertrophy, and surgical procedures involve resection, ablation or crushing of part, or all, of the turbinate to increase the size of the nasal airway (37). Among these approaches, submucosal resection involves preservation of the mucosa and bone with reduction of the submucosal tissue (37). A common technique involves using a number 15 blade to make an incision in the anterior head of the inferior turbinate and then a powered instrument, such as a small microdebrider can be introduced to resect the submucosa. The theory behind submucosal resection is that it preserves the ciliary function and mucociliary clearance by protecting the mucosa, but removes the tissue that is hypertrophied and leads to nasal obstruction (37). In our case, fine calcified material was densely deposited in the submucosa of the nasal cavity over a wide area, making it difficult to completely resect the lesion; thus, we decided to perform debulking surgery to improve the symptoms. In the operation, we tried to resect and reduce the submucosal tissues of the nasal septum and inferior turbinate where the amyloid material was deposited using a small microdebrider; however, but the calcified material was too tightly deposited to be reduced. Therefore, we attempted to reduce the calcified submucosal tissue and preserve the surface of the mucosa as much as possible using a UBC. A UBC is a metal instrument with a vibrating tip that was developed to resect bone tissue (38). It has the following features: it can specifically resect hard tissues (e.g., bone) while preserving soft tissues (e.g., dura mater) using the difference in natural frequencies; and it has no rotating parts, so it does not penetrate into the surrounding vital structures or pull structures into the axis of rotation (38). The UBC permits precise graded removal of bone without damage to the surrounding nasal soft tissue or mucosa (39). Previous studies have reported the applications of the UBC in various otorhinolaryngological surgeries to prevent accidental injuries to the adjacent soft tissue or vital structures (40–48). The use of UBC in septoplasty and turbinoplasty, submucosal dissection of septal spur, bony septum, and bone of inferior turbinate, and was associated with a good prognosis and fewer surgical complications in comparison of the use of a microdebrider (40, 44, 48). In our case, we used a UBC to resect diffuse calcified submucosal deposits of amyloid protein instead of dissection of the septal or turbinate bone, preserving as much of the mucosal surface as possible. There were no major complications associated with the use of UBC, such as nasal septal perforation, bleeding, empty nose syndrome or severe atrophic rhinitis. The UBC could be a useful tool for debulking surgery for sinonasal amyloidosis, especially in patients with extensive diffuse submucosal calcified lesions, such as our case, and in patients with lesions adjacent to vital structures, such as the skull base, orbit, or optic nerve.

Treatment for systemic AL amyloidosis includes chemotherapy to control clonal plasma cell dyscrasia and to decrease the synthesis of amyloidogenic light chains to reduce the progressive damage to amyloidotic organs (49). For decades, the treatment of systemic AL amyloidosis has centered on alkylating agent-based therapy with high-dose melphalan and autologous stem cell transplantation (ASCT). Within the framework of clinical trials, the disease has most likely been treated in accordance with the risk stratification of the standard Mayo Clinic staging system (6). Low-risk patients receive ASCT with melphalan (200 mg/m^2^) (6). Induction therapy with cyclophosphamide, bortezomib, and dexamethasone should be considered if bone marrow, plasma cell infiltration is >10% or if the patient refuses transplantation. Post-transplant bortezomib increases the complete response rate, and if a complete response is not achieved, combination therapy with bortezomib and dexamethasone should be administered (6). In intermediate-risk patients, combination therapy with melphalan and dexamethasone is preferred, especially if neuropathy or *t*_(11,14)_ translocation is present (6). Cyclophosphamide combined with bortezomib and dexamethasone is a stem cell-sparing regimen and is suitable for patients with renal failure and increased 1q21 (6). The combination of bortezomib, melphalan, and dexamethasone is preferred if the noninvasive free light chain level is >180 mg/l (6). In high-risk patients, bortezomib—which has a faster onset of action—may be preferred; however, low-dose combination therapy may also be preferred (6). In contrast to systemic AL amyloidosis, both localized and systemic chemotherapy for localized amyloidosis have been shown to be ineffective (10). In our review of the literature, one case was treated with systemic chemotherapy with melphalan and dexamethasone, which was also ineffective (30), suggesting that chemotherapy may be also ineffective for localized sinonasal amyloidosis.

Radiotherapy targeting the presumed clonal proliferation seems to be a useful treatment method for localized amyloidosis and is effective for stopping the progression of disease and improving symptoms caused by localized amyloidosis (9). In localized laryngeal or tracheobronchial amyloidosis, radiotherapy with or without debulking surgery has been performed for local control of progressive legions and was associated with good results (50–55). Among the cases identified in our review of the literature on localized sinonasal amyloidosis, only one case was treated with radiotherapy (36 Gy), which achieved good local control and good symptomatic improvement (31). In this case, the amyloid lesions had destructive extension to the bilateral frontal and anterior ethmoid sinuses, nasal cavity, and nasal bones, and were judged to be inoperable. Thus, radiotherapy seems to be an effective treatment for cases of sinonasal amyloidosis with inoperable extension in order to prevent the further progression of lesions. The appropriate dose of radiotherapy to treat localized amyloidosis has not yet been established (56). In the case of airway amyloidosis, doses of 20–45 Gy have been reported (50–55). Further studies are required to determine the adequate dose or scheduling of radiation therapy for localized sinonasal amyloidosis.

## Conclusion

We presented an extremely rare case of localized, idiopathic, primary nasal AL amyloidosis. Based on our review of the relevant literature, localized sinonasal amyloidosis shows a slow progression of nonspecific symptoms, such as nasal obstruction, epistaxis, and rhinorrhea, over a period of several years, and non-specific findings, such as mass formation in the nose or thickening of the mucosa; thus, clinicians should always suspect the presence of amyloidosis. The presence of adhesion of the nasal mucosa in the absence of previous surgery is a clue to the suspicion of amyloidosis. The only way to diagnose amyloidosis is to demonstrate amyloid deposits in the tissue by biopsy. CT often shows a fluffy appearance, which is an osteogenic reaction adjacent to sinonasal bone, or a large degree of punctuate calcification in soft tissue. MRI shows low to moderate signal intensity on T1-weighted images and variable signal intensity, ranging from low to high, on T2-weighted images. Chemotherapy is not effective for localized sinonasal amyloidosis, and the first-line treatment is resection or debulking of the lesion without impairing the sinonasal function; however, long-term follow-up is necessary because recurrence is frequently observed after treatment.

## Data Availability Statement

The original contributions presented in the study are included in the article/supplementary material, further inquiries can be directed to the corresponding author/s.

## Ethics Statement

The studies involving human participants were reviewed and approved by Ethics Committee, Toyama University Hospital. The patients/participants provided their written informed consent to participate in this study.

## Author Contributions

HTak, HTac, KT, and HS: conception and design. HTak, HTac, KT, and JI: literature search and obtaining of images. HTac, JI, and HS: writing the article. All authors critical revision and final approval of the article.

## Conflict of Interest

The authors declare that the research was conducted in the absence of any commercial or financial relationships that could be construed as a potential conflict of interest.

## Publisher's Note

All claims expressed in this article are solely those of the authors and do not necessarily represent those of their affiliated organizations, or those of the publisher, the editors and the reviewers. Any product that may be evaluated in this article, or claim that may be made by its manufacturer, is not guaranteed or endorsed by the publisher.

## Abbreviations

AA: amyloid derived from serum amyloid A protein
AL: amyloid derived from the immunoglobulin light-chain
ASCT: autologous stem cell transplantation
ATTR: amyloid derived from the transport protein transthyretin
CBC: Complete blood count
CT: computed tomography
MRI: magnetic resonance imaging
UBC: ultrasonic bone curette.
